# Supplementary material for: Snapin promotes HIV‐1 transmission from dendritic cells by dampening TLR8 signaling
Source: EMBO J. 2017 Oct 16;36(20):2998–3011. doi: 10.15252/embj.201695364 (PMC5641917; doi:10.15252/embj.201695364)
Supplement: Supplementary file 1 — Appendix [file EMBJ-36-2998-s001.pdf]

Appendix

Snapin promotes H IV-1 transmission from dendritic cells by dampening TLR8 signalling

Elham Khatamzas[1*], Madeleine Maria Hipp[1*], Daniel Gaughan[1], Tica Pichulik[1], Alasdair Leslie[1], Ricardo Fernandes[1], Daniele Muraro[1], Sarah Booth[1], Kieran Zausmer[1], Mei-Yi Sun[1], Benedikt Kessler[2], Sarah Rowland-Jones[1], Vincenzo Cerundolo[1], Alison Simmons[1, 3]†.

[1] MRC Human Immunology Unit, Weatherall Institute of Molecular Medicine, Oxford University, UK

[2] Henry Wellcome Building for Molecular Physiology, Department of Clinical Medicine, Oxford University

[3] Translational Gastroenterology Unit, Nuffield Department of Medicine, Oxford

Appendix Index

Page 2. Appendix Figure S1. Off-gel fractionation reduces the complexity of the phosphoproteome.

Page 2. Appendix Figure S2. Internalization of HIV-1 in Snapin knockdown cells.

Page 2. Appendix Figure S3. Immunoblot of Flag-tagged Snapin expression in DCs.

Page 3. Appendix Figure S4. Mean intensity of Rab7 expression in Ctrl or Snapin knockdown cells.

Page 3. Appendix Figure S5. qPCR of HIV-1 early expressed genes.

Page 3. Appendix Figure S6. TLR8 modulates induction of TNFα by HIV-1 in Snapin knockdown cells.

Pages 4-6. Appendix Table S1. Proteins exclusively phosphorylated in HIV-1 infected fractions.

Page 7. Appendix Table S2. Proteins exclusively phosphorylated in mock infected fractions.

Page 8. Appendix Table S3. Proteins dephosphorylated in HIV-1 infected fractions compared to mock infected fractions.

Page 9. Appendix Table S4. Proteins phosphorylated in HIV-1 infected fractions compared to mock infected fractions.

Page 10. Appendix Table S5. HIV-1 signalosome overlap with HIV-1 human interaction database.

Page 11. Appendix Table S6. Molecules identified within the HIV-1 signalosome that were in the top five canonical pathways in Ingenuity Pathway Analysis.

Pages 12-13. Appendix Table S7. List of genes tested in RNAi screen for effect on HIV-1 *trans*-infection.

Appendix Figure S1: Off-gel fractionation reduces the complexity of the phosphoproteome. Phosphoprotein-enriched samples of DCs mock infected or exposed to HIV-1 for 10 min were separated into 12 fractions by isoelectric focusing, desalted and subjected to electrophoresis. Silver stain of each fraction (F1-F12) is shown.

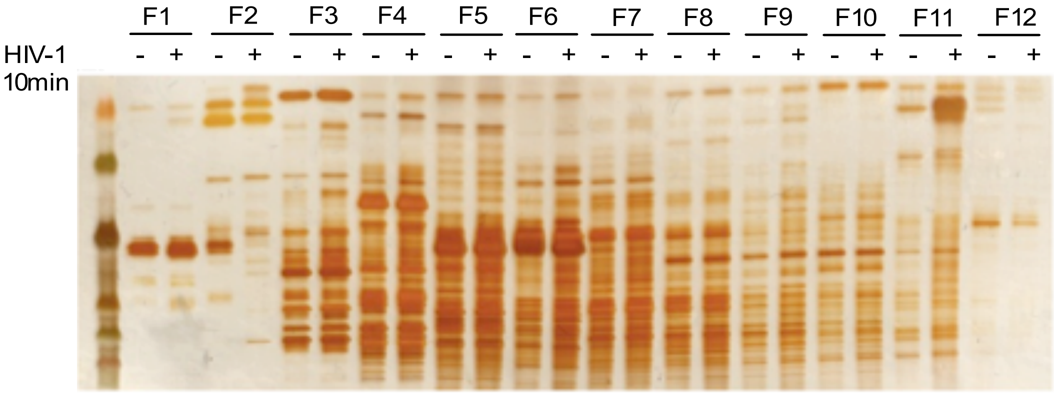

Appendix Figure S2. DCs transfected with Ctrl, Snapin siRNA or siRNAs targeting other genes represented on the siRNA screen examining *trans*-infection assessing the effect of these genes on internalization of HIV-1 into DCs. Internalization of ATTO 488 labelled HIV in DCs was measure by FACS at 2 hrs.

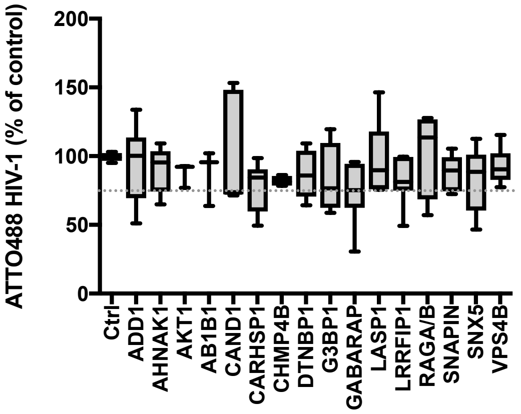

Appendix Figure S3. DCs transfected with Ctrl, WT, V92 and L99 Snapin-flag vectors. Immunoblot using anti-Flag antibody shown.

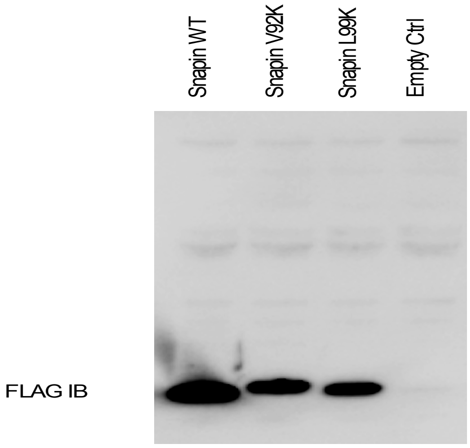

Appendix Figure S4. Mean intensity value of Rab7 staining in Ctrl or Snapin siRNA transfected cells.

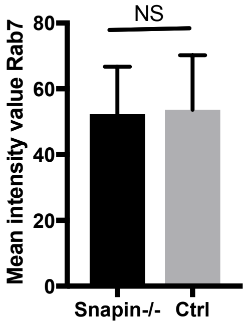

Appendix Figure S5. qPCR for early expressed HIV-1 genes tat-rev in Ctrl or Snapin siRNA transfected DCs exposed to HIV-1 at 6 hrs. Transfected DCs were infected for 2 hrs with HIV-1, washed and mRNA was harvested after 6 hrs. Values are relative to GAPDH. Data are representative of 3 experiments.

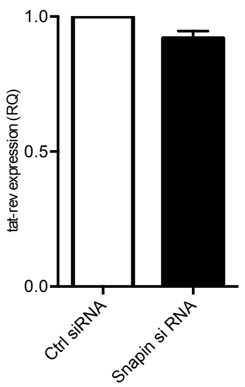

Appendix Figure S6. TLR8 modulates induction of TNFα by HIV-1 in Snapin knockdown cells. Thp1 were used as these cells have a macrophage like phenotype and have been shown to serve as a good model for studying TLR8 signaling pathways. Thp1 were matured with PMA/IL-4 to induce functional DC-SIGN expression. Thp1 were infected with Ctrl or Snapin shRNAs and then transfected with Ctrl or TLR8 siRNAs. Cells were either mock or HIV-1 infected prior to analysis of TNFα levels.

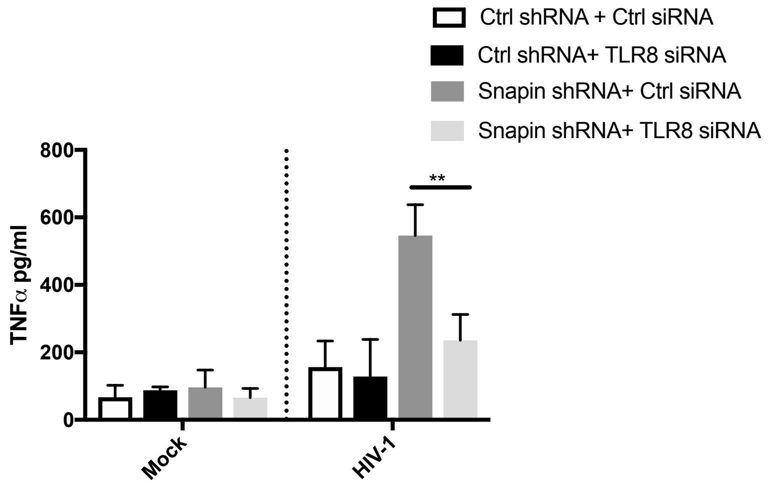

Appendix Table S1

Table 1: Proteins exclusively phosphorylated in HIV-infected fractions

Protein ID	Gene Symbol	Protein Name	Peptides Used	Spectra Used
IPI00382470	HSP90AA1	Isoform 1 of Heat shock protein HSP 90-alpha	30	606
IPI00010779	TPM4	Isoform 1 of Tropomyosin alpha-4	27	503
IPI00419258	HMGB1	HMGB1 High mobility group protein B1	15	149
IPI00008530	RPLP0	60S acidic ribosomal protein P0	9	35
IPI00021405	LMNA	LMNA Progerin	9	11
IPI00013894	STIP1	STIP1 protein	9	9
IPI00335168	MYL6B	Isoform Non-muscle of Myosin light polypeptide 6	8	86
IPI00470498	SERBP1	Isoform 3 of Plasminogen activator inhibitor 1 RNA-binding protein	7	20
IPI00003925	PDHB	PDHB 35 kDa protein	7	8
IPI00033494	MYL12B	Myosin regulatory light chain	5	13
IPI00059242	SYAP1	Synapse-associated protein 1	5	8
IPI00479722	PSME1	Proteasome activator complex subunit 1 isoform 2	5	7
IPI00215884	SFRS1	Isoform ASF-3 of Splicing factor, arginine/serine-rich 1	4	6
IPI00182938	AHCYL1	Adenosylhomocysteinase	4	5
IPI00291016	NDUFV3	Isoform 2 of NADH dehydrogenase [ubiquinone] flavoprotein 3, mitochondrial	4	5
IPI00106642	SDF2L1	Dihydropyrimidinase-like 2	4	5
IPI00218830	NMT1	Isoform Long of Glycylpeptide N-tetradecanoyltransferase 1	4	4
IPI00419979	PAK2	Serine/threonine-protein kinase PAK 2	4	4
IPI00015856	DNPEP	DNPEP 52 kDa protein	4	2
IPI00179330	UBB	Ubiquitin	3	9
IPI00300585	RRAGC	Ras-related GTP-binding protein C	3	6
IPI00012442	G3BP1	Ras GTPase-activating protein-binding protein 1	3	6
IPI00024971	OSBP	Isoform 1 of Oxysterol-binding protein 1	3	6
IPI00063234	PRKAR2A	cAMP-dependent protein kinase type II-alpha regulatory subunit	3	5
IPI00465028	TPI1	Isoform 1 of Triosephosphate isomerase	3	4
IPI00453476	PGAM1	Phosphoglycerate mutase 1	3	3
IPI00184670	AMPD2	Isoform Ex1B-2-3 of AMP deaminase 2	3	3
IPI00034319	CUTA	Isoform B of Protein CutA	3	2
IPI00465132	COPE	Coatomer subunit epsilon	2	12
IPI00101095	C20orf27	C20orf27 hypothetical protein LOC54976	2	11
IPI00009057	G3BP2	Isoform B of Ras GTPase-activating protein-binding protein 2	2	9
IPI00008527	RPLP1	60S acidic ribosomal protein P1	2	9
IPI00008994	NDRG2	Isoform 5 of Protein NDRG2	2	8
IPI00299033	KPNA3	Importin subunit alpha-3	2	7
IPI00023704	LPP	Lipoma-preferred partner	2	5
IPI00298558	PDCD10	Programmed cell death protein 10	2	4
IPI00643152	HSPA1L	HSPA1L 64 kDa protein Tax	2	4
IPI00021766	RTN4	Isoform 5 of Reticulon-4	2	4
IPI00105620	RRAGB	Isoform 2 of Ras-related GTP-binding protein B	2	4
IPI00384051	PSME2	Putative uncharacterized protein PSME2	2	4
IPI00473047	PRKAG1	5'-AMP-activated protein kinase subunit gamma-1 isoform 2	2	3
IPI00100160	CAND1	Isoform 2 of Cullin-associated NEDD8-dissociated protein 1	2	3
IPI00012535	DNAJA1	DnaJ homolog subfamily A member 1	2	3
IPI00002212	STK24	FLJ61383, highly similar to Serine/threonine-protein kinase 24	2	2
IPI00456359	ATXN2L	Ataxin-2-like protein isoform E	2	2
IPI00019927	PSMD7	26S proteasome non-ATPase regulatory subunit 7	2	2
IPI00215965	HNRNPA1	Isoform 2 of Heterogeneous nuclear ribonucleoprotein A1	2	2
IPI00867509	CORO1C	Coronin-1C	2	2

Appendix Table S1 continued

Table 1: Proteins exclusively phosphorylated in HIV-infected fractions

IPI00216917	C22orf9	Chromosome 22 open reading frame 9	2	2
IPI00472164	WASF2	Wiskott-Aldrich syndrome protein family member 2	2	2
IPI00221222	SUB1	Activated RNA polymerase II transcriptional coactivator p15	2	2
IPI00216393	CLTA	Isoform Non-brain of Clathrin light chain A	2	2
IPI00012578	KPNA4	Importin subunit alpha-4	2	2
IPI00012837	KIF5B	Kinesin-1 heavy chain	2	1
IPI00101987	C19orf62	Isoform 3 of BRCA1-A complex subunit MERIT40	2	1
IPI00027269	CBL	E3 ubiquitin-protein ligase CBL	2	1
IPI00031423	KRT33B	KRT33B 46 kDa protein	2	0
IPI00177716	HMGA1	HMGA1 protein	1	18
IPI00555621	PAK2	p21-activated kinase 2 variant (Fragment)	1	18
IPI00008219	RAD23A	UV excision repair protein RAD23 homolog A	1	5
IPI00011937	PRDX4	Putative uncharacterized protein PRDX4	1	4
IPI00289758	CAPN2	cDNA FLJ58224, highly similar to Calpain-2 catalytic subunit	1	4
IPI00027107	TUFM	Tu translation elongation factor, mitochondrial precursor	1	4
IPI00007426	ARL6IP5	cDNA FLJ52128, highly similar to PRA1 family protein 3	1	4
IPI00027253	GABARAP	Gamma-aminobutyric acid receptor-associated protein	1	3
IPI00022145	NUCKS1	Isoform 2 of Nuclear ubiquitous casein and cyclin-dependent kinases substrate	1	3
IPI00059764	ZNF428	Isoform 1 of Zinc finger protein 428	1	3
IPI00219678	EIF2S1	Eukaryotic translation initiation factor 2 subunit 1	1	3
IPI00304409	CARHSP1	Calcium-regulated heat stable protein 1	1	3
IPI00000675	CNPY3	Isoform 1 of Protein canopy homolog 3	1	2
IPI00019901	ADD1	Alpha-adducin isoform d	1	2
IPI00000634	CCDC6	Coiled-coil domain-containing protein 6	1	2
IPI00010860	PSMD9	Isoform p27-L of 26S proteasome non- ATPase regulatory subunit 9	1	2
IPI00000792	CRYZ	crystallin, zeta (quinone reductase)	1	2
IPI00302592	FLNA	Filamin A, alpha	1	2
IPI00183526	NCL	NCL 32 kDa protein	1	2
IPI00005064	GPSM3	G-protein- signaling modulator 3	1	2
IPI00465439	ALDOA	Fructose-bisphosphate aldolase A	1	2
IPI00025084	CAPNS1	Calpain small subunit 1	1	2
IPI00018229	PLDN	Isoform1 of Pallidin	1	2
IPI00008575	KHDRBS1	cDNA FLJ54590, highly similar to KH domain-containing, RNA-binding, signaltransduction-associated protein 1	1	2
IPI00027272	BRCA1	BRCA1 25 kDa protein	1	2
IPI00431127	CCNK	Isoform 3 of Cyclin K	1	2
IPI00012503	PSAP	Isoform Sap-mu-6 of Proactivator polypeptide	1	2
IPI00003419	C11orf58	C11orf58 Small acidic protein	1	2
IPI00008289	BRCC3	BRCA1/BRCA2-containing complex, subunit 3	1	2
IPI00296485	MAP1S	Microtubule-associated protein 1S	1	2
IPI00302850	LOC100129492	Similar to small nuclear ribonucleoprotein D1 polypeptide 16kDa	1	2
IPI00007765	HSPA9	cDNA FLJ51903, highly similar to Stress-70 protein, mitochondrial	1	1
IPI00012833	PPP4C	Serine/threonine-protein phosphatase 4 catalytic subunit	1	1
IPI00396435	DHX15	Putative pre-mRNA-splicing factor ATP-dependent RNA helicase	1	1
IPI00003084	DRAP1	DR1-associated co-repressor	1	1
IPI00025807	SP3	Transcription factor Sp3 isoform 3	1	1
IPI00375380	PSMD13	Isoform 2 of 26S proteasome non- ATPase regulatory subunit 13	1	1
IPI00024740	YAF2	YY1 associated factor 2	1	1
IPI00169383	PGK1	Phosphoglycerate kinase	1	1
IPI00216699	FERMT3	Isoform 1 of Fermitin family homolog 3	1	1
IPI00011107	IDH2	Isocitrate dehydrogenase	1	1
IPI00028059	NUMB	Isoform 3 of Protein numb homolog	1	1

Appendix Table S1 continued

Table 1: Proteins exclusively phosphorylated in HIV-infected fractions

IPI00023191	TOM1	cDNA FLJ54710, highly similar to Target of Myb protein 1	1	1
IPI00639863	MTM1	Myotubularin	1	1
IPI00784154	HSPD1	60 kDa heat shock protein, mitochondrial	1	1
IPI00291412	PPM1F	Protein phosphatase 1F	1	1
IPI00003527	SLC9A3R1	Na(+)/H (+) exchange regulatory cofactor NHE-RF1	1	1
IPI00301034	NADK	NAD kinase	1	1
IPI00020602	CSNK2A2	Casein kinase II subunit alpha	1	1
IPI00152695	WDR82	WD repeat-containing protein 82	1	1
IPI00288941	NCOA5	Nuclear receptor coactivator 5	1	1
IPI00550821	CPSF7	Isoform 1 of Cleavage and polyadenylation specificity factor subunit 7	1	1
IPI00059292	MAGOHB	Protein mago nashi homolog 2	1	1
IPI00291175	VCL	cDNA FLJ53006, highly similar to Vinculin	1	1
IPI00171176	PANK2	Isoform 4 of Pantothenate kinase 2, mitochondrial	1	1
IPI00304692	RBMX	cDNA FLJ34201 fis, clone FCBBF3019714, highly similar to heterogenou nuclear ribonucleoprotein G	1	1
IPI00289601	HDAC2	Isoform 1 of Histone deacetylase 2	1	1
IPI00017964	SNRPD3	Small nuclear ribonucleoprotein Sm	1	1
IPI00021088	KCNAB1	cDNA FLJ59247, highly similar to Voltage-gated potassium channel subu beta-1	1	1
IPI00018465	CCT7	T-complex protein 1 subunit eta isoform c	1	1
IPI00293857	ARRB1	Isoform 1A of Beta-arrestin-1	1	1
IPI00018398	PSMC3	26S protease regulatory subunit 6A	1	1
IPI00219483	SNRNP70	Isoform 1 of U1 small nuclear ribonucleoprotein 70 kDa	1	1

Appendix Table S2

Table 2: Proteins exclusively phosphorylated in mock-infected fractions				
**Protein ID**	**Gene Symbol**	**Protein Name**	**Peptides Used**	**Spectra Used**
IPI00414676	HSP90AB1	Heat shock protein HSP 90-beta	34	248
IPI00382894	TPM3	Tropomyosin 3, isoform CRA_b	18	194
IPI00007750	TUBA4A	TUBA4A cDNA FLJ58687, highly similar to Tubulin alpha-4 chain	10	35
IPI00789605	MYL6	Isoform Smooth muscle of Myosin light polypeptide 6	9	35
IPI00651653	DDX17	Isoform 3 of Probable ATP-dependent RNA helicase DDX17	5	11
IPI00396378	HNRNPA2B1	Isoform B1 of Heterogeneous nuclear ribonucleoproteins A2/B1	3	4
IPI00003865	HSPA8	Isoform 1 of Heat shock cognate 71 kDa protein	3	2
IPI00167419	ANKRD44	Isoform 1 of Serine/threonine-protein phosphatase 6 regulatory ankyrin repeat subunit B	2	3
IPI00011268	RALY	RNA binding protein, autoantigenic	2	3
IPI00183046	PTPN6	Isoform 3 of Tyrosine-protein phosphatase non-receptor type 6	2	3
IPI00031023	FLII	Protein flightless-1 homolog	2	2
IPI00304925	HSPA18	cDNA FLJ54408, highly similar to Heat shock 70 kDa protein 1	2	2
IPI00218993	HSPH1	Isoform Alpha of Heat shock protein 105 kDa	2	2
IPI00297169	LCP2	Lymphocyte cytosolic protein 2	2	1
IPI00010397	HLA-DRB1	HLA-DRB1	2	1
IPI00010320	CBX1	Chromob2x protein homolog 1	1	3
IPI00010133	CORO1A	Coronin-1 A	1	2
IPI00011454	GANAB	Isoform 3 of Neutral alpha-glucosidase AB	1	2
IPI00339277	SAMSN1	SAMSN1 Isoform 3 of SAM domain-containing protein SAMSN-1	1	2
IPI00012433	F8A1	Factor VIII intron 22 protein	1	2
IPI00171199	PSMA3	Isoform 1 of Proteasome subunit alpha type-3	1	1
IPI00009123	NUCB1	Isoform 2 of Nucleobindin-2	1	1
IPI00015973	EPB41L2	EPB41L2 band 4.1-like protein 2 isoform b	1	1
IPI00026670	TCEB2	Transcription elongation factor B (SIII), polypeptide 2 (18kDa, elongin B), isoform CRA_b	1	3
IPI00394804	RINL	Ras and Rab interactor-like protein	1	1
IPI00298994	TLN1	Talin 1	1	1
IPI00030355	PPP1R11	Protein phosphatase 1 regulatory subunit 11	1	1
IPI00027378	UBXN1	Isoform 2 of UBX domain-containing protein 1	1	1
IPI00166137	RALYL	RNA-binding Raly-like protein isoform 1	1	1
IPI00739464	LOC646821	LOC646821 similar to actin, gamma 1	1	1
IPI00296353	ARHGAP18	Isoform 2 of Rho GTPase-activating protein 18	1	1
IPI00183274	SNX1	cDNA FLJ46302, highly similar to Sortin nexin 1	1	1
IPI00064767	ARHGAP17	Isoform 1 of Rho GTPase-activating protein 17	1	1
IPI00220365	EIF4G1	EIF4G1 protein	1	1
IPI00647217	SKIV2L2	Superkiller viralicidic activity 2-like 2	1	1
IPI00013949	SGTA	Small glutamine-rich tetratricopeptiderepeat-containing protein A	1	1
IPI00332936	ZC3HAV1	Isoform 2 of Zinc finger CCCH-type antiviral protein 1	1	1
IPI00007244	MPO	Isoform H7 of Myeloperoxidase	1	1
IPI00012866	AKT1	RAC-alpha serine/threonine-protein kinase	1	1
IPI00009634	SQRDL	Sulfide:quinone oxidoreductase, mitochondrial	1	1
IPI00788612	LIMS1	LIM and senescent cell antigen-like-containing domain protein 1	1	1
IPI00005613	LOC441722	Similar to U2 small nuclear RNA auxillary factor 1	1	1
IPI00000816	YWHAE	14-3-3 protein epsilon	1	1
IPI00470477	ATG4B	Putative uncharacterized protein ATG4B	1	1
IPI00020319	BLOC1S1	Isoform 1 of Biogenesis of lysosome- related organelles complex 1 subunit 1	1	3
IPI00329338	PCYT1A	Choline-phosphate cytidylyltransferase A	1	1

Appendix Table S3

Table 3: Proteins dephosphorylated in HIV-1-infected fractions compared to mock-infected fractions

Gene Symbol	Length (AA)	Peptides Used	Spectra Used	HIV Norm SI	Mock Norm SI	Ratio	Log2
HSPB1	205	6	16	1.82E-05	2.77E-07	0.02	-6.03
ATP5A1	553	4	6	9.16E-07	3.88E-08	0.04	-4.56
EEF1D	281	5	10	1.22E-05	5.40E-07	0.04	-4.5
HNRNPU	806	7	37	5.20E-06	2.30E-07	0.04	-4.5
ST13	369	3	6	4.32E-06	1.92E-07	0.04	-4.49
PSMB7	277	1	1	1.36E-07	6.19E-09	0.05	-4.46
HNRNPC	293	5	13	8.00E-06	4.43E-07	0.06	-4.17
PEA15	130	2	9	1.99E-05	1.27E-06	0.06	-3.97
CANX	627	2	4	5.18E-07	3.75E-08	0.07	-3.79
EEF2	858	11	17	1.62E-06	1.29E-07	0.08	-3.65
MYH9	1960	10	12	7.11E-07	7.02E-08	0.1	-3.34
GAPDH	335	2	2	7.52E-07	1.01E-07	0.13	-2.89
OSTF1	214	3	5	9.09E-06	1.29E-06	0.14	-2.82
VASP	380	10	35	1.39E-05	2.00E-06	0.14	-2.79
PRKAR1A	381	4	8	9.86E-06	1.75E-06	0.18	-2.49
SNX5	404	3	5	2.03E-06	4.16E-07	0.2	-2.29
C6orf108	174	1	2	2.77E-06	5.98E-07	0.22	-2.21
EEF1B2	225	1	3	7.36E-06	1.62E-06	0.22	-2.19
HSD17B4	736	7	8	7.06E-07	1.62E-07	0.23	-2.13
FAM129B	733	15	66	1.44E-05	3.65E-06	0.25	-1.98
RCC2	522	9	22	6.82E-06	1.76E-06	0.26	-1.95
BSCL2	747	6	9	2.64E-06	7.44E-07	0.28	-1.83
AKAP12	1684	1	54	1.61E-05	4.73E-06	0.29	-1.76
RPS6	249	2	2	6.05E-07	1.84E-07	0.3	-1.72
EIF2S3	472	13	34	8.60E-06	2.85E-06	0.33	-1.59
SSR4	184	1	1	2.00E-07	6.99E-08	0.35	-1.52
ACLY	1101	9	12	1.10E-06	3.94E-07	0.36	-1.49
CTPS	591	1	2	2.53E-07	9.08E-08	0.36	-1.48
ENO3	434	1	2	3.64E-07	1.34E-07	0.37	-1.45
PSMA7	248	12	46	4.68E-05	1.74E-05	0.37	-1.42
PDIA4	645	9	21	4.29E-06	1.61E-06	0.37	-1.42
LOC645870	89	1	4	2.48E-05	9.33E-06	0.38	-1.41
ATG7	703	5	3	2.44E-07	9.20E-08	0.38	-1.41
PSMB4	264	5	19	8.72E-06	3.29E-06	0.38	-1.41
STK4	487	1	3	7.77E-07	2.97E-07	0.38	-1.39
PLEK	350	8	38	1.58E-05	6.08E-06	0.39	-1.38
PDAP1	181	1	1	5.30E-07	2.10E-07	0.4	-1.33
IRF2BP2	587	4	3	4.78E-07	1.93E-07	0.4	-1.31
HDGF	240	7	16	2.73E-05	1.12E-05	0.41	-1.28
ZYX	485	1	2	5.29E-07	2.22E-07	0.42	-1.25
S100A9	114	3	56	1.87E-04	8.07E-05	0.43	-1.21
BCKDK	382	7	22	8.49E-06	3.71E-06	0.44	-1.2
PSMB5	263	1	4	8.36E-07	3.83E-07	0.46	-1.13
AHNAK	5890	2	2	3.50E-08	1.61E-08	0.46	-1.12
CFL1	166	8	47	3.09E-05	1.57E-05	0.51	-0.97
KCTD12	325	4	4	2.59E-07	1.42E-07	0.55	-0.87
CALR	417	17	161	3.32E-04	1.95E-04	0.59	-0.76
ALOX15	684	4	3	3.37E-07	1.98E-07	0.59	-0.76
PSMA4	261	6	23	1.17E-05	7.18E-06	0.61	-0.71
EEF1G	487	5	5	8.94E-07	5.52E-07	0.62	-0.69
PDIA3	505	3	4	9.49E-07	5.90E-07	0.62	-0.69
HNRNPK	463	3	4	7.37E-07	4.60E-07	0.62	-0.68
CAP1	475	10	53	1.49E-05	9.45E-06	0.63	-0.66
HSPA5	655	14	23	1.77E-05	1.12E-05	0.64	-0.65
YBX1	324	4	1	1.49E-06	9.49E-07	0.64	-0.65
ACTB	375	6	13	2.06E-05	1.34E-05	0.65	-0.63
PPP1R7	360	4	11	5.86E-06	3.82E-06	0.65	-0.62
PSMB1	241	9	25	2.21E-05	1.44E-05	0.65	-0.62
NASP	788	4	5	4.26E-07	2.86E-07	0.67	-0.57
NPM1	265	4	10	1.54E-05	1.05E-05	0.68	-0.55
MIR1279	551	1	6	4.46E-07	3.05E-07	0.68	-0.55
F13A1	732	5	16	3.92E-06	2.72E-06	0.69	-0.53
NAP1L1	391	5	9	5.11E-06	3.61E-06	0.71	-0.5
LCP1	627	26	104	6.76E-05	4.84E-05	0.72	-0.48
SSB	408	6	7	1.44E-06	1.04E-06	0.72	-0.47
PALLD	672	3	4	4.97E-07	3.64E-07	0.73	-0.45

Appendix Table S4

Table 4: Proteins phosphorylated in HIV-1-infected fractions compared to mock-infected fractions

Gene Symbol	Length (AA)	Peptides Used	Spectra Used	HIV Norm SI	Mock Norm SI	Ratio	Log2
PSMB6	239	2	1	1.83E-08	4.14E-06	226.37	7.82
RCSD1	416	1	1	2.70E-09	2.38E-07	87.92	6.46
DR1	176	2	2	7.63E-07	1.92E-05	25.2	4.66
PRMT5	620	1	1	1.51E-08	2.11E-07	14.04	3.81
PDHA1	428	1	1	3.26E-07	3.95E-06	12.12	3.6
PCNP	178	2	1	1.10E-07	1.23E-06	11.16	3.48
SFRS3	164	3	5	9.85E-07	1.09E-05	11.05	3.47
PA2G4	394	1	1	1.40E-07	1.39E-06	9.91	3.31
HCLS1	486	2	2	7.90E-08	6.96E-07	8.8	3.14
ZNF737	536	1	10	1.14E-07	9.37E-07	8.2	3.04
NT5C2	561	1	3	2.34E-07	1.91E-06	8.18	3.03
SH3BP1	701	1	1	1.28E-07	9.06E-07	7.08	2.82
DPYSL2	619	1	1	7.42E-08	5.03E-07	6.78	2.76
GFPT1	699	1	2	7.14E-08	4.71E-07	6.59	2.72
LASP1	261	2	10	3.35E-06	2.14E-05	6.38	2.67
PSMB3	205	3	4	1.21E-06	6.62E-06	5.45	2.45
BCL2L13	485	2	1	1.49E-07	7.85E-07	5.27	2.4
APOB48R	1088	13	26	5.86E-06	3.07E-05	5.25	2.39
TALDO1	337	5	18	3.14E-06	1.53E-05	4.86	2.28
PSMA1	263	6	7	3.16E-06	1.52E-05	4.81	2.27
YWHAZ	245	2	3	1.04E-06	4.95E-06	4.74	2.24
PYGB	843	9	7	1.30E-06	5.92E-06	4.56	2.19
SET	290	6	28	1.71E-05	7.53E-05	4.39	2.13
SEPT9	422	6	2	5.53E-07	2.17E-06	3.92	1.97
EIF2S2	333	3	3	2.81E-07	1.10E-06	3.92	1.97
BTF3L4	158	1	4	3.17E-06	1.24E-05	3.91	1.97
PSMB2	201	4	5	2.25E-06	8.65E-06	3.83	1.94
SAMHD1	626	25	86	1.12E-05	4.27E-05	3.83	1.94
LRRFIP1	784	8	13	3.78E-06	1.44E-05	3.8	1.93
VIM	466	4	6	4.84E-07	1.80E-06	3.72	1.89
NACA2	215	2	1	3.82E-06	1.42E-05	3.7	1.89
LIMA1	759	2	1	6.20E-08	2.24E-07	3.6	1.85
MAP2K1	393	3	4	4.31E-07	1.52E-06	3.52	1.81
PSMA6	246	7	22	5.84E-06	1.92E-05	3.28	1.71
CDC37	378	2	3	2.62E-07	8.41E-07	3.21	1.68
EIF4B	616	1	5	6.44E-07	2.04E-06	3.16	1.66
VCP	806	2	3	3.08E-07	9.59E-07	3.11	1.64
P4HB	508	11	19	5.56E-06	1.72E-05	3.1	1.63
SEPT2	361	7	14	6.41E-06	1.97E-05	3.07	1.62
B2M	119	1	2	1.27E-06	3.62E-06	2.86	1.51
VPS4B	444	3	3	6.45E-07	1.80E-06	2.79	1.48
PRKCSH	535	13	37	1.65E-05	4.12E-05	2.5	1.32
CD74	296	2	2	4.46E-07	1.09E-06	2.45	1.29
NACA	215	7	79	5.62E-05	1.36E-04	2.42	1.27
RNH1	461	6	5	8.34E-07	1.92E-06	2.3	1.2
RCN1	331	1	1	2.84E-07	6.53E-07	2.3	1.2
AP1B1	949	1	2	1.43E-07	3.25E-07	2.27	1.18
CSNK2A1	397	4	6	1.70E-06	3.73E-06	2.19	1.13
PSMB8	276	6	15	5.01E-06	1.07E-05	2.13	1.09
PKM2	531	3	6	6.48E-07	1.34E-06	2.07	1.05
HIST1H4F	103	1	1	1.42E-06	2.84E-06	2	1
S100A8	93	4	24	1.88E-05	3.59E-05	1.91	0.94
MARCKS	332	9	24	6.47E-05	1.20E-04	1.86	0.89
LSP1	339	15	141	1.87E-04	3.26E-04	1.74	0.8
TFG	400	1	1	2.94E-07	4.85E-07	1.65	0.72
ILF2	390	4	6	2.96E-06	4.70E-06	1.59	0.67
CORO1B	489	4	5	7.44E-07	1.14E-06	1.53	0.61
RPLP2	115	6	49	2.37E-04	3.50E-04	1.48	0.56
HMGB2	209	11	36	2.33E-05	3.43E-05	1.47	0.55
PTMS	102	1	2	2.27E-06	3.24E-06	1.43	0.51
BTF3	206	2	3	2.27E-06	3.16E-06	1.39	0.48
MSN	577	19	89	2.07E-05	2.86E-05	1.39	0.47
MVD	400	2	2	4.29E-07	5.65E-07	1.32	0.4
PSMA2	234	4	9	4.19E-06	5.49E-06	1.31	0.39
PURA	322	1	2	2.39E-07	3.06E-07	1.28	0.35
C1QBP	282	7	24	2.64E-05	3.35E-05	1.27	0.35
ILF3	690	7	13	1.51E-06	1.91E-06	1.26	0.34
DBNL	439	2	1	3.46E-07	4.33E-07	1.25	0.33

Appendix Table S5

Table 5: HIV-1 signalosome overlap with HIV-1 human interaction database

Proteins exclusively phosphorylated in HIV-infected fractions	Proteins exclusively phosphorylated in mock-infected fractions	Proteins dephosphorylated in HIV-1-infected fractions compared to mock-infected fractions	Proteins phosphorylated in HIV-1-infected fractions compared to mock-infected fractions
AMPD2	EIF4G1	ACLY	AP1B1
CAPN2	EPB41L2	ACTB	BTF3
CCNK	GANAB	ATG7	C1QBP
FLNA	HLA-DRB1	ATP5A1	CD74
G3BP1	HNRNPA2B1	CALR	CDC37
G3BP2	HSP90AB1	CANX	CSNK2A1
GABARAP	HSPA8	CFL1	DPYSL2
HDAC2	HSPH1	CTPS	DR1
HMGB1	LCP2	EEF1D	EIF4B
HNRNPA1	MPO	EEF1G	HIST1H4F
HSP90AA1	MYL6	EEF2	HMGB2
HSPA1L	PSMA3	EIF2S3	ILF2
HSPA9	SGTA	F13A1	ILF3
HSPD1	SKIV2L2	GAPDH	LIMA1
IDH2	SQRDL	HDGF	LSP1
KHDRBS1	TCEB2	HNRNPC	MAP2K1
KIF5B	TLN1	HNRNPK	MARCKS
KPNA3	TUBA4A	HNRNPU	MSN
KPNA4	YWHAE	HSD17B4	NACA
LMNA	ZC3HAV1	HSPA5	P4HB
MAP1S		HSPB1	PA2G4
MTM1		LCP1	PDHA1
MYL12B		MYH9	PKM2
MYL6B		NAP1L1	PRKCSH
NCL		NASP	PSMA1
NMT1		NPM1	PSMA2
NUCKS1		PDAP1	PSMA6
PAK2		PDIA3	PSMB2
PDHB		PDIA4	PSMB3
PGK1		PRKAR1A	PSMB6
PPM1F		PSMA4	PSMB8
PRDX4		PSMA7	PTMS
PRKAG1		PSMB1	PURA
PRKAR2A		PSMB4	RCN1
PSMC3		PSMB5	RNH1
PSMD13		PSMB7	S100A8
PSMD7		RCC2	SAMHD1
PSMD9		RPS6	SEPT9
PSME1		SSR4	SET
PSME2		SSB	SFRS3
RAD23A		YBX1	VCP
RBMX		ZYX	VIM
RPLP0			VPS4B
RTN4			YWHAZ
SDF2L1			
SFRS1			
SLC9A3R1			
SNRNP70			
SNRPD3			
SP3			
STIP1			
SUB1			
TOM1			
TPI1			
TUFM			
UBB			
VCL			
WASF2			
WDR82			

Appendix Table S6. Molecules identified within the HIV-1 signalosome that were in the top five canonical pathways in Ingenuity Pathway Analysis.

**Pathway**	**Molecules**
**Protein ubiquitination**	B2M, BRCA1, CBL, DNAJA1, HSP90AA1, HSP90AB1, HSPA8, HSPA9, HSPA1A/HSPA1B, HSPA1L, HSPB1, HSPD1, HSPH1, PSMA1, PSMA3, PSMA6, PSMA7, PSMB2, PSMB3, PSMB4, PSMB5, PSMB6, PSMB7, PSMB8, PSMC3, PSMD7, PSMD9, PSMD13, PSME1, PSME2, TCEB2, UBB
**Amyloid processing**	AKT1, CAPN2, CAPNS1, CSNK2A1, CSNK2A2, PRKAG1, PRKAR1A, PRKAR2A
**Antigen presentation**	B2M, CANX, CD74, HLA-DRB1, PSMB5, PSMB6, PSMB8
**Glycolysis/Gluconeogenesis**	ALDOA, ENO3, GAPDH, PDHA1, PDHB, PGAM1, PGK1, PKM2, TPI1
**IGF1 signaling**	AKT1, CSNK2A1, CSNK2A2, MAP2K1, PRKAG1, PRKAR1A, PRKAR2A, YWHAE, YWHAZ

Appendix Table S7. List of genes tested in RNAi screen for effect on HIV-1 *trans*-infection. DCs were transfected with Smartpool siRNAs for indicated genes and control non-silencing siRNA, harvested 48hrs later and infected with NL4-3 BaL for 2 h, subsequently co-cultured with $CD4^+$ PHA-blasts for 72 hours before analysis for intracellular p24 gag in CD4+ T cells. Values represent *trans*-infection as measured by intracellular p24 gag in $CD4^+$ T cells relative to siRNA control. Values represent mean of 3-4 biological replicates $\pm$ SEM. * denotes double knockdown of RRagA+RRagB and RRagC+RRagD, respectively.

Gene ID	>100%	76-100%	50-75%	25-49%
ADD1				**0.45 (0.11)**
ADD3			**0.59 (0.10)**	
AHCYL1	1.08 (0.10)			
AHNAK			**0.64 (0.08)**	
AKAP12		0.90 (0.20)		
AKT1			**0.69 (0.22)**	
ALOX15		0.88 (0.01)		
AP1B1			**0.58 (0.21)**	
ARL6AP5		0.83 (0.16)		
ARRB1		0.92 (0.23)		
ATG4B		0.90 (0.11)		
ATG7		0.76 (0.04)		
BAIAP2		0.97 (0.15)		
BLOC1S1		0.81 (0.13)		
BRCA1		0.92 (0.29)		
BRCC3			**0.60 (0.14)**	
CAND1				**0.44 (0.18)**
CAPNS1		0.82 (0.19)		
CARHSP1				**0.49 (0.01)**
CBL20		0.93 (0.26)		
CD74			**0.56 (0.19)**	
CHMP4B			**0.65 (0.16)**	
CLTA			**0.75 (0.15)**	
CNO		0.94 (0.15)		
CNPY3			**0.74 (0.15)**	
COPE	1.12 (0.30)			
CORO1B			**0.70 (0.18)**	
CTPS		0.77 (0.12)		
DBNL	1.04 (0.15)			
DPYSL		0.93 (0.12)		
DR1			**0.62 (0.10)**	
EIF2S1		0.83 (0.11)		
EIF4B		0.82 (0.19)		
EIF4G1			**0.74 (0.17)**	
ENO2			**0.73 (0.05)**	
EPB41L2	1.08 (0.05)			
EVL	1.1 (0.12)			
EZR		0.85 (0.03)		
FERMT3		0.80 (0.21)		
FLII		0.87 (0.04)		
FLNA			**0.75 (0.12)**	
G3BP1			**0.61 (0.18)**	
G3BP2			**0.72 (0.22)**	
GABARAP			**0.68 (0.1)**	
GC1QR		0.78 (0.21)		
GFPT1			**0.75 (0.04)**	
HCLS1		0.98 (0.23)		
HSD17B4		0.94 (0.25)		
HSPA8		0.90 (0.12)		
HSPB1			**0.66 (0.11)**	
KIAA1949		0.76 (0.07)		
KIF5B	1.04 (0.27)			
KPNA4			**0.70 (0.19)**	
LASP1			**0.60 (0.06)**	
LCP2	1.26 (0.25)			
LIMA1		0.79 (0.16)		
LIMS1			**0.54 (0.14)**	
LPP			**0.74 (0.17)**	
LRRFIP1			**0.63 (0.06)**	
MAP1S			**0.72 (0.07)**	
MAP2K1			**0.75 (0.03)**	

Gene ID	>100%	76-100%	50-75%	25-49%
MARCKS			**0.63 (0.12)**	
MSN			**0.61 (0.03)**	
MST		0.85 (0.10)		
MVD	1.48 (0.25)			
NCL			**0.61 (0.17)**	
NDRG2			**0.63 (0.08)**	
NPM1			**0.62 (0.16)**	
NUCB1			**0.73 (0.20)**	
NUMB	1.26 (0.28)			
OSBP			**0.70 (0.07)**	
OSTF1		0.94 (0.08)		
PA2G4	1.25 (0.30)			
PALLD		0.84 (0.16)		
PASP			**0.71 (0.24)**	
PCNP			**0.56 (0.21)**	
PEA15		0.90 (0.20)		
PEX19		0.95 (0.41)		
PLDN		0.94 (0.08)		
PLEK			**0.65 (0.03)**	
PPP1R7	1.05 (0.17)			
PPP4C		0.98 (0.06)		
PRKAR2A		0.85 (0.06)		
PRMT5		0.92 (0.18)		
PSAP			**0.71 (0.24)**	
PSMB6			**0.75 (0.2)**	
RAD23A		0.78 (0.20)		
RCC2	1.05 (0.16)			
RCN1		0.84 (0.03)		
RCSD1	1.07 (0.16)			
RINL		0.87 (0.22)		
RPS6		0.83 (0.14)		
RRAGA*			**0.68 (0.06)**	
RRAGC*	1.18 (0.25)			
S100A8		0.95 (0.05)		
S100A9	1.02 (0.32)			
SAMHD1	1.11 (0.21)			
SAMSN1			**0.72 (0.05)**	
SFRS3			**0.66 (0.06)**	
SHP-1	1.15 (0.25)			
SLC9A3R1	1.10 (0.20)			
SNRNP70	1.17 (0.13)			
SNX1		0.96 (0.05)		
SNX5			**0.75 (0.24)**	
SSB			**0.65 (0.12)**	
SSR4		0.92 (0.11)		
TCEB2		0.96 (0.12)		
TFG		0.98 (0.15)		
TLN1			**0.64 (0.06)**	
TOM1			**0.63 (0.04)**	
TUBA4A		0.81 (0.18)		
VASP			**0.70 (0.15)**	
VCL		0.82 (0.06)		
VCP			**0.70 (0.03)**	
VIM		0.95 (0.14)		
VPS4B				**0.40(0.07)**
WAVE2		0.82 (0.01)		
YBX1			**0.71 (0.17)**	
ZC3HAV1			**0.66 (0.18)**	
ZYX			**0.69 (0.09)**	
