# Supplementary figures and images for: Snapin promotes HIV‐1 transmission from dendritic cells by dampening TLR8 signaling
Source: EMBO J. 2017 Oct 16;36(20):2998–3011. doi: 10.15252/embj.201695364 (PMC5641917; doi:10.15252/embj.201695364)

Appendix Figure S3

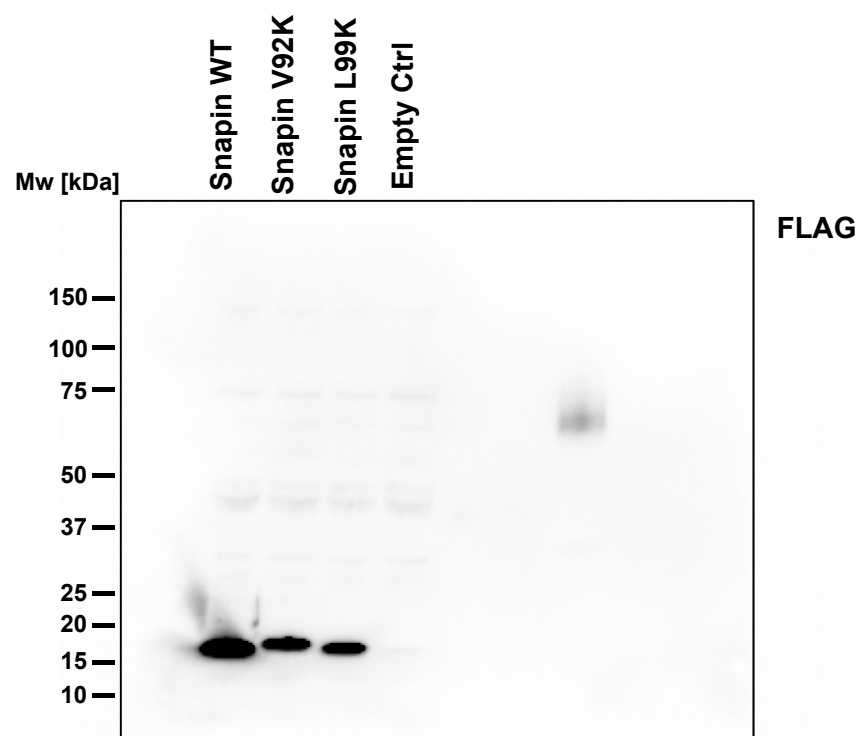

Supplement: Supplementary file 2 — Source Data for Appendix [file EMBJ-36-2998-s005.zip › SourceDataAppendixFigS3.pdf]

Fig 1A

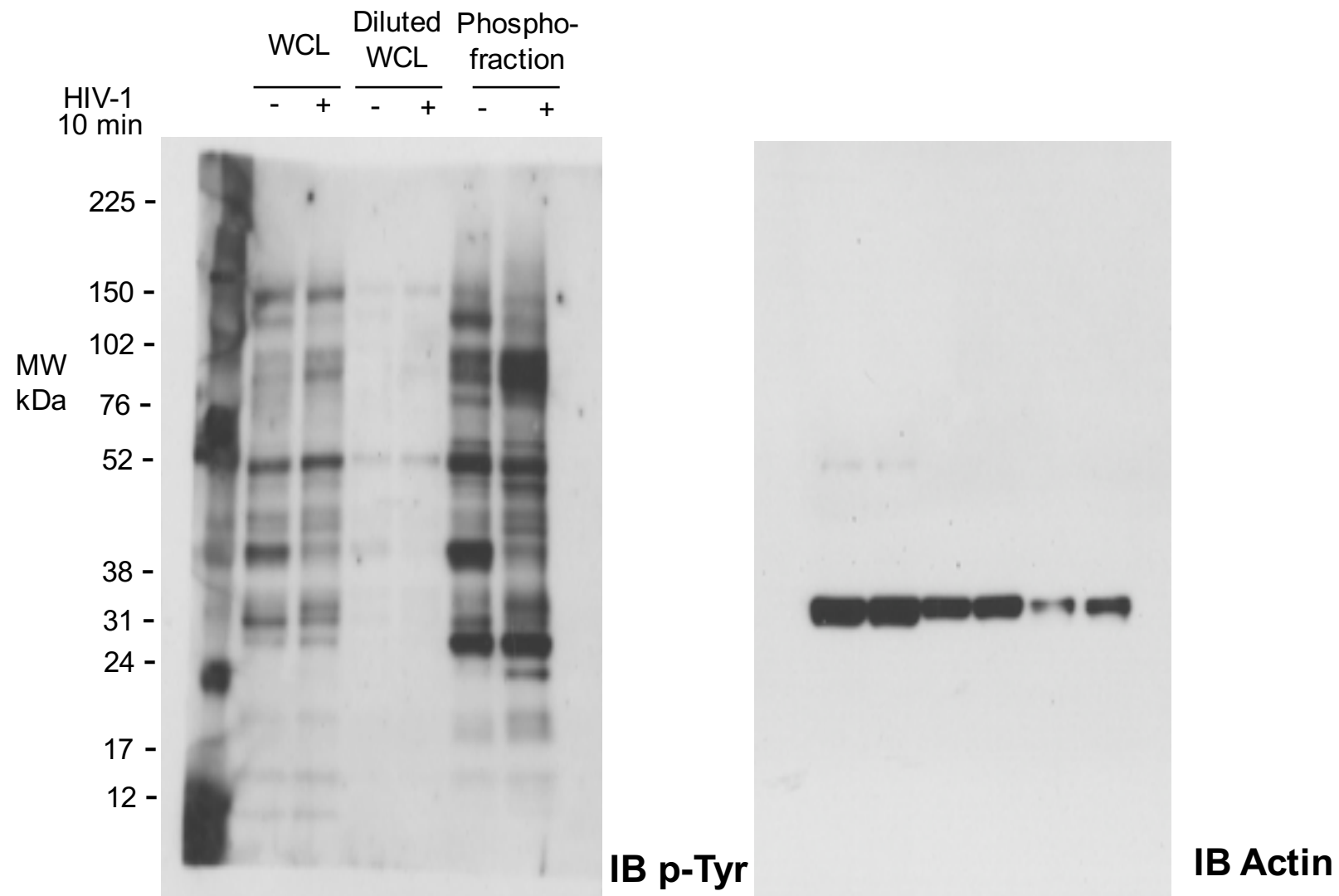

Supplement: Supplementary file 4 — Source Data for Figure 1A [file EMBJ-36-2998-s002.pdf]

### Figure 4A

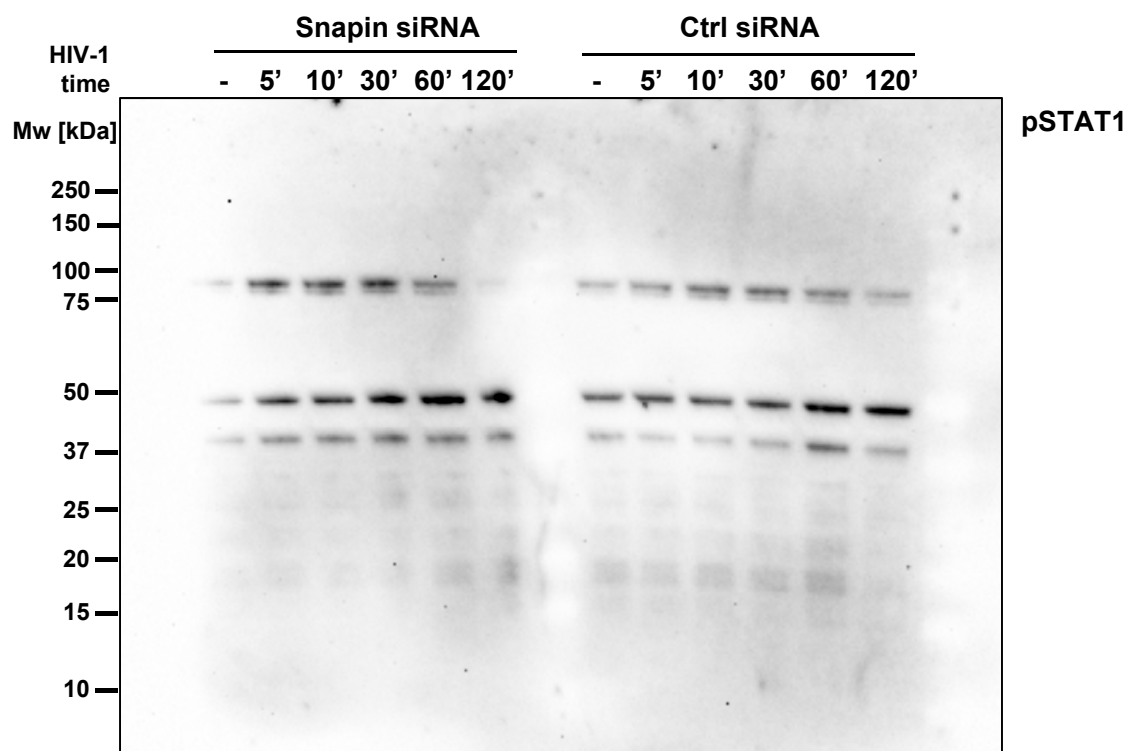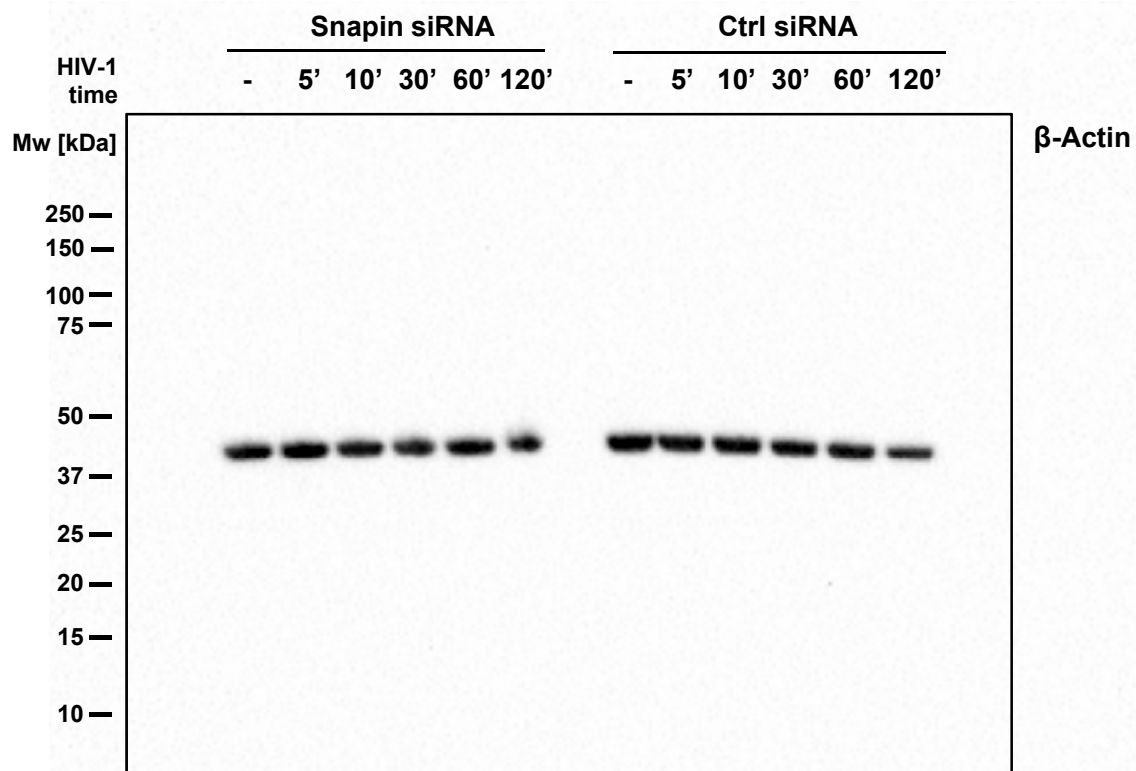

Supplement: Supplementary file 5 — Source Data for Figure 4A [file EMBJ-36-2998-s003.pdf]

Figure 5C

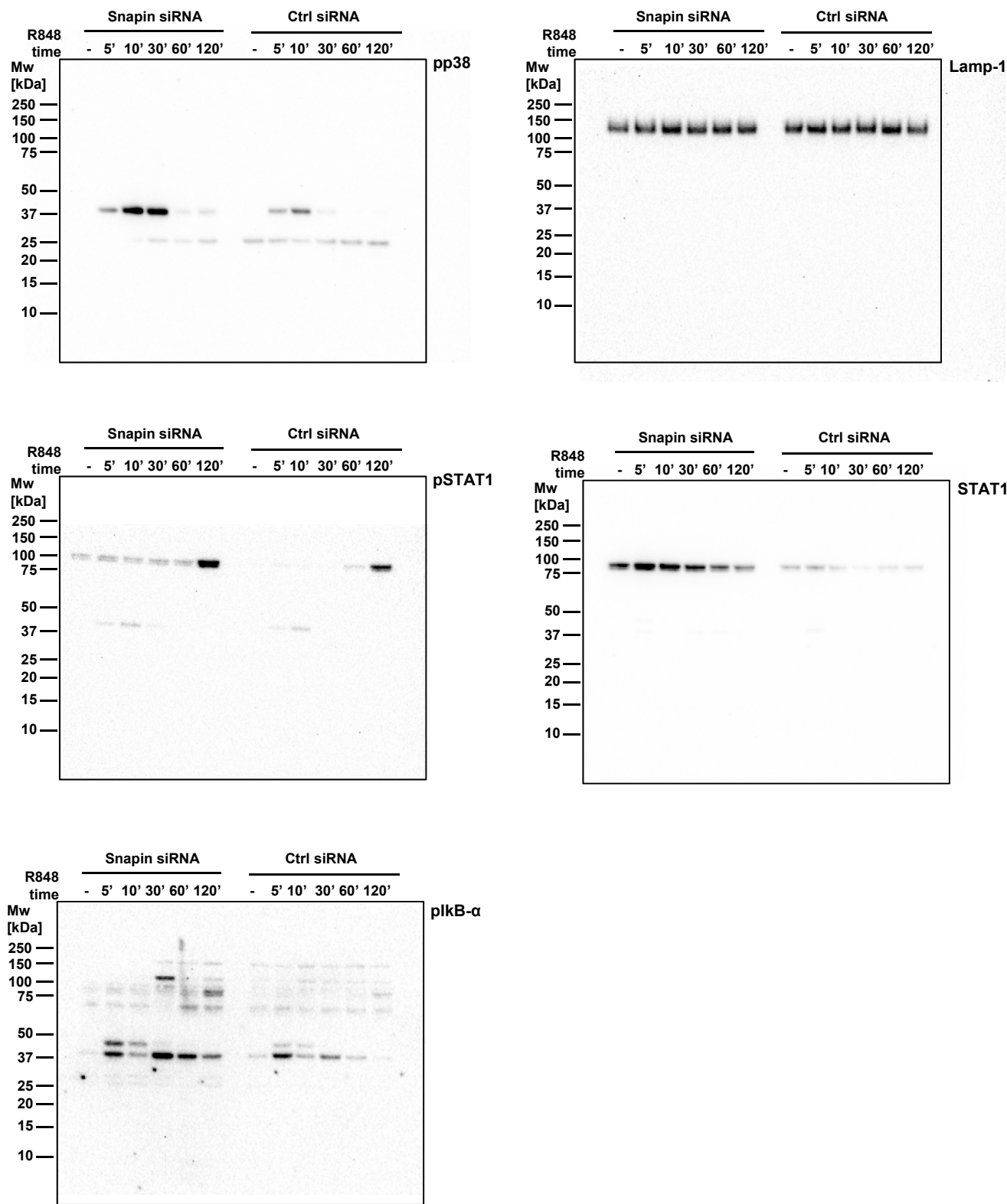

Supplement: Supplementary file 6 — Source Data for Figure 5C and D [file EMBJ-36-2998-s004.zip › embj201695364-sup-0000-SDataFig5C.pdf]

Figure 5D

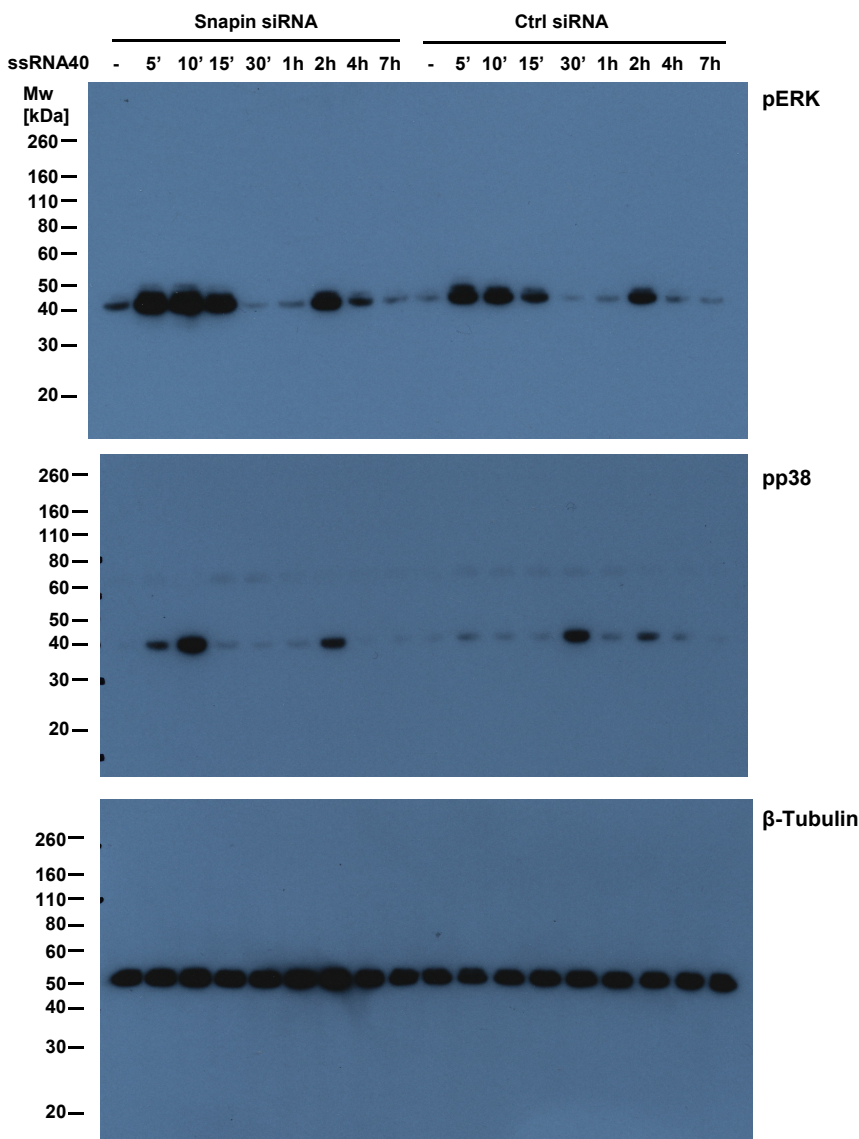

Supplement: Supplementary file 6 — Source Data for Figure 5C and D [file EMBJ-36-2998-s004.zip › embj201695364-sup-0000-SDataFig5D.pdf]
